# Not all anosmia and fever was COVID-19 infection: a case report

**DOI:** 10.11604/pamj.2020.37.371.27014

**Published:** 2020-12-23

**Authors:** Niamien Patrice Koffi, Cherif El Asri Abad, Gazzaz Miloud, El Mostarchid Brahim

**Affiliations:** 1 Neurosurgery Department, Mohamed V Military Training Hospital, Rabat, Morocco

**Keywords:** Anosmia, fever, COVID-19, fronto-ethmoid meningioma, case report

## Abstract

COVID-19 pandemic touch all part of world to the date more than fifteen millions of patients are infected by virus including about 1,388,926 deaths (European Centre for Disease Prevention and Control an agency of the European Union). Morocco has put in place strict containment measures to control the disease and prevent the saturation of health systems. One of the great difficulties is to quickly identify asymptomatic and paucisymptomatic cases which function as an important vector of contagion. Anosmia and fever are one of revealed mode for the young patient but is not all the case. We report one case in the sense. A 40-year-old man without medical history was admitted in the hospital after complaining 3 days ago clinical symptoms of fever, cough, headache and anosmia. Immediately, the patient benefits of COVID-19 protocol, measure of fever, nasal swab and polymerase chain reaction (PCR) test. Despite the negativity of PCR test of COVID-19, the patient was placed in isolation. Two days later, he presented a generalized seizures, then we performed a cerebral computed tomography scan (CT scan) which showed a bilateral frontal oedema. The cerebral magnetic resonance imaging (MRI) revealed the presence of 4x4x4 cm well enhanced meningeal extra axial mass of the anterior skull base with peri-tumoral oedema corresponding to an olfactory groove meningioma. The tumour was totally resected through a left fronto-lateral approach. The postoperative courses were uneventful with the persistence of totally anosmia.

## Introduction

The COVID-19 pandemic linked to the SARS-CoV-2 virus is a major public health problem. To date more than fifteen millions of patients are infected by virus including about 1,388,926 deaths (European Centre for Disease Prevention and Control an agency of the European Union). Morocco looks like the whole planet has put in place strict containment measures to control the disease and prevent the saturation of health systems. One of the great difficulties is to quickly identify asymptomatic and pauci-symptomatic cases which function as an important vector of contagion. Hyposmia or anosmia and fever are one of relative led mode for the young patient. Several series around the world (China, South Korea, Iran, Europe and the United States) indicate a partial or total loss of smell and/or taste in 20 to 90% of patients with SARS-CoV-2 [1-3]. But isn´t all the case, we report an observation in this sense.

## Patient and observation

A 40-year-old Moroccan patient, living in Tangier province, without any medical history. Admitted from a provincial hospital for headache with influenza-like illness plus anosmia evolving for 3 days .The history of its symptoms reveals a recent flu syndrome associated with a loss of smell and cough in the context of the COVID-19 epidemic. Patient suspected of a case of COVID-19 is placed under isolation and the PCR and the nasal swab taken. During his isolation he had two episodes of convulsion with vomiting. After stopping the convulsive seizures with medical treatment, he was evacuated in our hospital (Military Hospital of Rabat).

Medical examen in our hospital. The interrogation found a progressive decrease in progressive visual acuity. With two episodes of vomiting and partial seizures with recent secondary generalization in a context of influenza like illness. The admission clinic exam; after the barrier measurements and temperature measurement, it is placed in isolation pending the result of the COVID-19 PCR and the sampling of the nasal swab. Secondary after the negative result of the PCR, the second clinical examination founded a conscious patient, Glascow coma scale=15/15, temperature is 37.8 °C, and his neck is flexible. Neurogically standing and walking are possible and the examen of muscle strength is normal to the 4 limbs. The osteotendinous reflexes are also normal and the cutaneo plantar reflexes are in bilateral flexion. The examination of the cranial nerves reveals anosmia indicating damage to the first pair of cranial nerves. The visual acuity was preserved in both eyes. The visual field in bed shows an anomaly of the bilateral nasal visual field. The fundus shows grade 1 papillary oedema.

**Figure 1:**
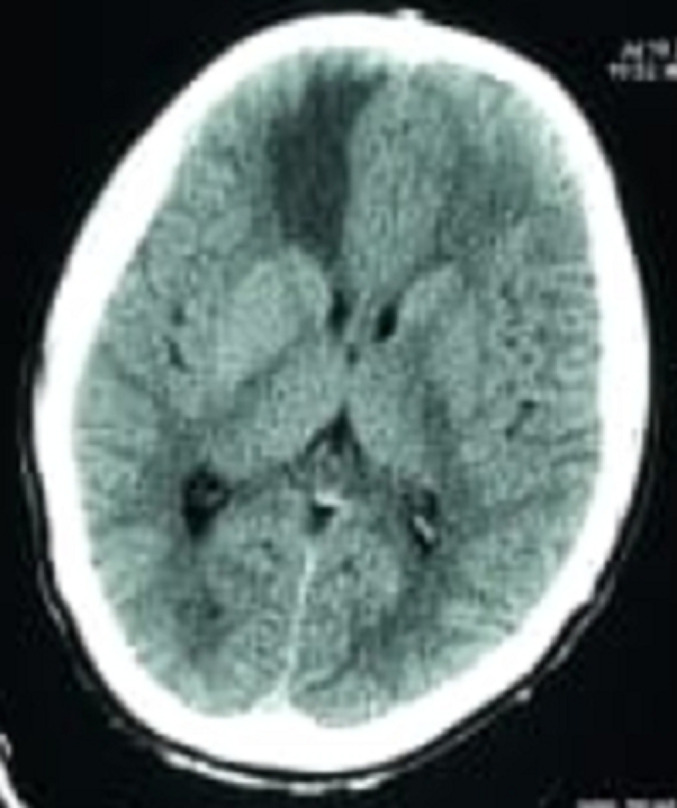
CT scan of a slightly rounded hyper density process of the extra-axial front anterior skull base sitting at the olfactory groove area with significant hypo density peri-lesional oedema on the toe finger more marked on the right and hyperostosis in regard of the lesion

**Figure 2:**
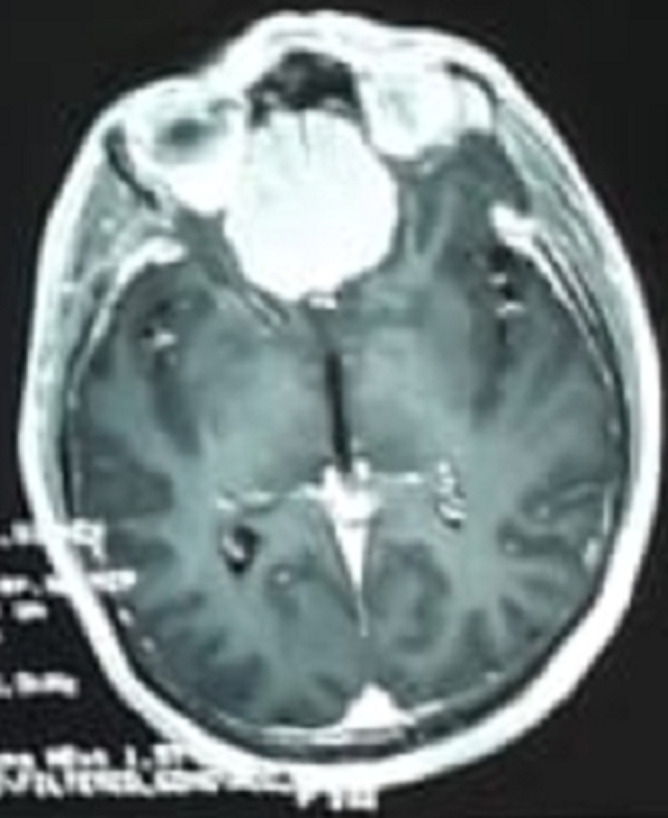
MRI scan of T1 lesion hypo intensity extra axial anterior skull base fronto ethmoidal area; in T2 lesion hyper intensity this lesion was homogenous inhancing after gadolinium injection

Pulmonary auscultation shows fine crackles in the lung bases in a eupneic patient with 98% saturation in ambient air. The rest of the somatic examination is unremarkable, in particular cardiac, digestive and cutaneous. It's a case of anosmia plus secondary epilepsy associated with influenza-like illness in a 40-year-old patient during COVID-19 pandemic. The biological assessment shows a protein reactive C=45 with 11,000 white blood cells with predominance of polynucleosis. The chest X-ray is normal. Then we performed a cerebral CT scan (Figure 1) which show a slightly rounded hyper-density process of the extra axial front floor sitting at the olfactory groove area with significant hypo-density peri-lesional oedema on the toe finger more marked on the right and hyper-osterosis in regard of the lesion. Secondary the cerebral MRI scan (Figure 2) revelead the presence 4x4x4 cm well enhanced meningioma extra axial mass of the anterior skull base with peri-tumoral oedema corresponding to an olfactory groove meningioma. Patient was prepared and operated. The tumour was totally resected through a left fronto-lateral approach. The postoperative courses were un-eventful with the persistence of totally anosmia.

## Discussion

In South Korea about 30% of SARS-CoV-2 positive patients had anosmia as the main symptom of COVID-19 [4]. The number of isolated anosmia without nasal obstruction or rhinorrhea is increasing [1,4-7]. This sudden anosmia could be an inaugural symptom of SARS-CoV-2 infection and thus be an early warning sign. Rather it affects young patients and is often associated with a favourable prognosis of the disease [5,6]. However, the lack of information in severely SARS-CoV-2 patients does not support this. These smell disorders occur either before the onset of general and ear-nose-throat (ENT) symptoms (12% of cases) or during (65% of cases) or after (23% of cases). In 20% of cases, anosmia is not associated with ear-nose-throat (ENT) symptoms (nasal obstruction or rhinorrhea). Women seem more affected (92 vs 82 %) [5].

Even in a pandemic period guided by symptomatology, physicians should still be able to have a wide range of differential diagnoses. Like chronic rhino-sinusitis, the most frequent, of post infectious, post-traumatic, neurodegenerative and idiopathic origin. In our case, it is a slowly evolving fronto-ethmoidal lesion process compressing the first cranial nerves responsible for secondary anosmia. The pathophysiological mechanism of anosmia due to SARS-CoV-2 infection is unknown. Several hypotheses exist. The loss of smell could be due to inflammation of the nasal mucosa or more targeted damage to the olfactory neuro-epithelium. Neurological damage via the olfactory pathways to certain brain areas and potentially to the brainstem are discussed [8,9]. The hematogenous dissemination of the virus in the central nervous system is also investigated. However, in the clinical picture of COVID-19, neurological damage is not in the foreground.

Entry of SARS-CoV-2 into the human host cell would use the same receptors as SARS-CoV-2 [10,11]. SARS-CoV-2 infection is thought to be related to the expression of two receptors on the target cell (ACE2 and PMPRSS2), as is the case with hair cells in the airways. Interestingly, a group of Geneva researchers has demonstrated that these receptors are also expressed at the level of the olfactory neuroepithelium, more particularly on the sustentacular cells (cells supporting the olfactory sensory neurons) [12]. Outcome in case of COVID-19 is good, a European multicenter study reports 15-day recovery of smell in 44% of patients [5]. The outcome of anosmia in case of anterior skull base meningioma couldn´t be recovered like our case.

## Conclusion

Meningioma is an extra-parenchymal tumor usually developed at the expense of arachnoid cells. It´s 15-20% of all primary adult brain tumors. Twice as common in women, their incidence increases in the second half of life with a peak in the fifth decade. Fronto ethmoidal meningiomas represent 10% of all meningiomas [13]. They thus resound on the olfactory bulb but also on the underside of the frontal lobes. The anosmia is the revealed mode of ethmoid meningioma, this symptom is frequently to the young patient with COVID-19 in 30%-90% of the case. It also principal signs of olfactory groove meningioma [1-3].
